# Cancer, Inflammation, and Insights from Ayurveda

**DOI:** 10.1155/2012/306346

**Published:** 2012-07-04

**Authors:** Venil N. Sumantran, Girish Tillu

**Affiliations:** ^1^Department of Biotechnology, Indian Institute of Technology Madras, Chennai 600 036, India; ^2^Symbiosis School of Biomedical Sciences, Symbiosis International University, Pune 412115, India

## Abstract

A recent, exciting discovery relates to the concept of “shared pathology” between cancer and metabolic syndrome. One major pathway common to cancer and metabolic syndrome is chronic inflammation, which is a major driving force in carcinogenesis. Indeed, chronic inflammation precedes most cancers and is considered a “hallmark” of the neoplastic process. We discuss molecular and biochemical evidence which links diet, obesity, abnormal lipid metabolism, and type 2 diabetes mellitus with chronic inflammation. We also explain how each of these factors is linked with biochemical aberrations of carcinogenesis and the prevalence and risk of cancer. While there are reliable biomarkers for chronic inflammation, there are few markers for a mechanistic link between early inflammation and digestive disorders. Discovery of such a marker could lead to identification of a new subtype of patients with digestive disorders that predispose them to cancer and/or metabolic syndrome. In this context, we discuss the ayurvedic concept of “*Ama*” which is thought to be a toxic, proinflammatory waste-product of improper digestion. We then develop hypotheses and outline preclinical and clinical experiments designed to prove whether “*Ama*” can serve as a novel and reliable biomarker that links abnormal digestive status, with the onset of chronic inflammation.

## 1. Introduction

The vast amounts of data from the omics revolution has led to the concept of translational medicine, wherein well-documented biological discoveries are used to produce new drugs and medical devices. Another outcome of the omics revolution is “personalized medicine” for individual patients or subtypes of patients. Indeed, both translational and personalized medicine are “hot fields” for today's pharmaceutical industries, and the race to find “personalized drugs” for cancer patients is well underway. Notably, the identification of certain sub-types of breast cancer has already resulted in some degree of personalized treatment for these patients. However, there is a gap between the quantity of genomic information and our ability to interpret it. One critical review states that “without a truly robust mechanism for selecting personalized medicine-we will continue to see only incremental improvements. Therefore, it is now imperative that future clinical trials be designed with a plan to incorporate biomarker development” [1]. Thus, the experts acknowledge limitations in the fields of translational and personalized medicine. These limitations have significant impact on cancer treatment, since cancer is a complex, multifactorial, and heterogenous disease which requires innovative approaches in translational and personalized medicine.

### 1.1. Personalized Medicine and Ayurveda

Although personalized medicine is new to modern medicine, it is well established in *Ayurveda*, the traditional system of Indian medicine which is still being practiced. Ayurvedic physicians perform careful analysis of host-drug interactions in order to prescribe personalized drugs for each patient. We have discussed the sophisticated nature of Ayurvedic personalized medicine with two examples [2]. First, we explained how individuals with different sub-types of osteoarthritis can be treated with different drugs. Next, we illustrated the dynamic nature of Ayurvedic medicine by explaining how a sequence of personalized drugs can be used to treat different stages of asthma in a single patient [2]. Since there are limitations in the current research on personalized medicine, it is important to carefully evaluate Ayurvedic concepts and develop hypotheses which may lead to novel methods of cancer diagnoses and biomarker development.

### 1.2. “Shared Pathology” between Cancer and Metabolic Syndrome

A recent, exciting discovery is the concept of “shared pathology” between cancer and metabolic syndrome. Thus, Hirsch et al. found several similarities between gene signatures for cancer and gene expression signatures from inflammatory, cardiovascular, and gastrointestinal diseases. Many of the genes common to these diseases regulate lipid metabolism and cholesterol biosynthesis [3, 4]. One major pathway common to both these diseases is chronic inflammation [5]. In fact, chronic inflammation is now considered a critical “hallmark” of carcinogenesis [6], and the molecular and biochemical mechanisms linking inflammation, lipid metabolism, and cancer are the subject of intense research. Therefore, this article reviews evidence for the newly discovered links between inflammation, lipid metabolism, and cancer. We also discuss relevant Ayurvedic concepts and outline experimental approaches that can contribute towards the development of new biomarkers for chronic inflammation. Before we describe the “shared pathology” between cancer and metabolic syndrome, we explain how chronic inflammation develops and contributes to tumorigenesis. Indeed, chronic inflammation precedes most cancers and is associated with at least 20% of all cancers [5, 6].

## 2. Chronic Inflammation in Cancer

### 2.1. Generation and Maintenance of Chronic Inflammation in Tumors

**Figure 1:**
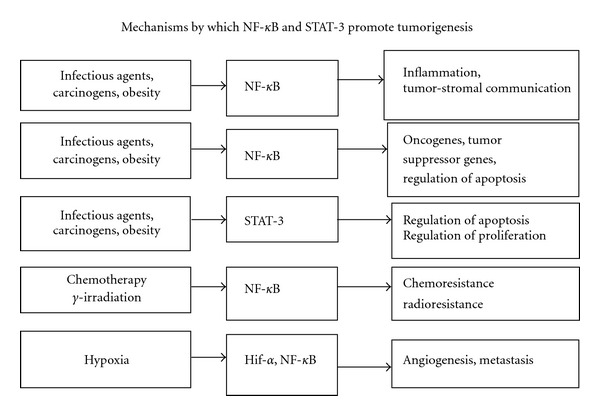
NF-*κ*B and STAT3 control inflammation and cancer. Nuclear factor-*κ*B (NF-*κ*B) and signal transducers and activators of transcription 3 (STAT3), are transcription factors which activate pathways causing inflammation, transformation, angiogenesis, and metastasis. NF-*κ*B and STAT3 are constitutively active in most cancers, and their suppression results in inhibition of tumor proliferation and invasion. Most chemopreventive agents act by inhibition of NF-*κ*B and STAT3 pathways.

 Short-term inflammation has anti-infective and anticancer effects, whereas prolonged or chronic inflammation can promote disease. Preclinical and clinical research, over the past decade, strongly suggests that chronic inflammation is associated with serious lifestyle and age-related diseases such as cancer and metabolic syndrome. Indeed, evidence also suggests that inflammatory microenvironment in and around tumors is an essential part of tumorigenesis. The molecular nature of the causal relationship between inflammation and cancer is only now being understood. Thus, there are two main ways in which the tumor microenvironment undergoes chronic inflammation. First, an intrinsic pathway of inflammation is driven by cells transformed by various genetic events (oncogenes, gene amplification, or inactivation of tumor-suppressor genes). In the second pathway, external inflammatory or infectious conditions increase the risk of developing cancer at certain sites (e.g., the colon, prostate, and pancreas) [5, 6]. These two pathways activate transcription factors such as nuclear factor-kappaB (NF-*κ*B), signal transducer and activator of transcription 3 (STAT-3), and hypoxia-inducible factor 1alpha (HIF-1 alpha), in tumor cells [6, 7]. Next, these transcription factors coordinate the overexpression, elevated secretion, or abnormal activation of proinflammatory mediators such as cytokines, chemokines, cyclooxygenase-2 (COX-2), prostaglandins, inducible nitric oxide synthase (iNOS), and nitric oxide. Inflammatory cells, such as tumor-infiltrating leukocytes and tumor associated macrophages (TAMs), are now recruited into the tumor stroma. TAMs in particular are considered prime regulators of cancer inflammation [5, 6]. The resulting inflammatory microenvironment directly promotes tumor progression by increasing tumor growth and survival, increasing evasion of apoptosis, and accelerating the processes of angiogenesis, invasion, and metastasis [5, 6].  Figure 1 summarizes the major tumor promoting effects of NF-*κ*B, STAT-3, and HIF-1 alpha.

### 2.2. Maintenance of Chronic Inflammation in Tumors

Once the inflammatory microenvironment has been created in tumors, there are mechanisms which sustain it. First, the cytokines which activated the transcription factors NF-*κ*B and STAT-3 in tumor cells also activate these same transcription factors in inflammatory cells and tumor-stromal cells, which in turn results in more inflammatory mediators being produced. Thus, the cancer-related inflammatory microenvironment is enhanced by activation of inflammation in cells surrounding the tumor. This sustained “smouldering” cancer-related inflammation has many tumor-promoting effects [5–7]. Another mechanism for maintaining the cancer-related inflammation involves inflammation induced by reactive oxygen and nitrogen species. These free radicals damage DNA, proteins, and lipids and result in gene mutation and accumulation of advanced glycation end products (AGE). Interaction of AGE with its receptor (RAGE) triggers chronic inflammation by activation of NF-*κ*B, at sites of tissue damage. The activated NF-*κ*B, overrides endogenous anti-inflammatory mechanisms and leads to sustained inflammation [7–9]. Indeed, the importance of the RAGE-AGE interaction was proved by showing that transgenic mice lacking RAGE protein, developed small skin tumors with low levels of pro-inflammatory mediators and reduced numbers of infiltrating immune cells, when compared with wild type mice. These data strongly suggest that the AGE-RAGE interaction is essential for maintaining the inflammatory microenvironment in tumors [8].

In addition to its direct tumor promoting effects, chronic inflammation can indirectly enhance tumor formation. For example, immunosuppression, which is a serious risk factor for initiation and promotion of tumors, results from chronic inflammation [5, 6]. Although the mechanisms are unknown, there is now good evidence that a chronic systemic inflammatory response results in progressive loss of weight in cancer patients, which is known as cachexia [10]. Thus, immunosuppression and cachexia are two important indirect effects of chronic inflammation on cancer.

### 2.3. Anti-Inflammatory Drugs for Cancer

Although our understanding of signaling pathways and transcription factors in chronic inflammation primarily comes from pre-clinical studies, these data have clinical relevance since certain anti-inflammatory drugs (such as nonsteroidal anti-inflammatory drugs (NSAIDs) and COX-2 specific inhibitors) have proven anticancer effects in pre-clinical tumor models and clinical trials. These anti-inflammatory drugs interfere with eicosanoid signaling and metabolism, suppress the neoplastic process, and can decrease oxidative stress and angiogenesis [11]. Interestingly, the potent antitumor effects of phytochemicals, such as curcumin, from turmeric, the green tea polyphenol epigallocatechin gallate (EGCG), and resveratrol from grapes, are in large part attributed to their anti-inflammatory activities [12]. Guggulsterone, the major active component of the gum resin from *Commiphora wightii* (or *Commiphora mukul*), is used to treat internal tumors, obesity, liver disorders, and malignant sores and ulcers in *Ayurveda*. Notably, guggulsterone was shown to induce apoptotic cell death and suppress proliferation, invasion, angiogenesis and metastasis of tumor cells [12]. Interestingly, all four phytochemicals (curcumin, EGCG, resveratrol, and guggulsterone) inhibit inflammation by suppressing the transcriptional activity of NF-*κ*B [12, 13]. In addition, guggulsterone may also inhibit the transcriptional activity of STAT-3 [13].

## 3. Cancer and Metabolic Syndrome

### 3.1. Abnormal Lipid Metabolism and Cancer

 Having explained the importance of chronic inflammation in tumor formation and progression, we review the evidence on the concept of “shared pathology” between cancer and metabolic syndrome [3, 4]. Metabolic syndrome refers to a cluster of related diseases (type 2 diabetes mellitus, cardiovascular disease, and obesity) which share abnormalities such as chronic inflammation and dyslipidemia. Therefore, in the next two sections, we discuss abnormalities of lipid metabolism in tumors, obesity, and how these processes lead to chronic inflammation.

Tumor cells metabolize large amounts of lipids, and lipid biosynthesis and desaturation of lipids are required for tumor cell survival. Indeed, enzymes, such as fatty acid synthase [14] and stearoyl-CoA desaturase [15], are often overexpressed in tumor cells and are potential targets for anticancer drugs. Cholesterol which serves as a precursor for the synthesis of many sex hormones has been linked to increased risk of prostate cancer [16]. However, the mechanisms which link cancer and cholesterol remains controversial, because antineoplastic therapies influence one's lipid profile, and anti-hyper lipidemic drugs, in turn, may influence the processes of malignancy [17]. However, recent data point to a direct link between Oxidized LDL receptor 1 (OLR-1) and cancer. Thus, OLR-1 may act as an oncogene, by activating the NF-*κ*B, which, in turn, induces expression of its target genes responsible for lipogenesis, cell proliferation, cell migration, inflammation, and inhibition of apoptosis [3, 18]. In addition, oxidized LDL itself can directly promote tumors by enhancing the generation of reactive oxygen species which damage and mutate DNA [19]. Oxidized LDL also indirectly promotes tumors by inducing proinflammatory changes in macrophages and reducing their phagocytic capacity towards dying tumor cells. In this context, it is important to note that oxidized LDL is generated in the body by several pathways [18, 19].

### 3.2. Obesity, Cancer, and Inflammation

 Epidemiological data have linked a high body mass index (BMI) and obesity in both genders to an enhanced risk of colorectal, esophageal, kidney cancer, non-Hodgkin's lymphoma, and multiple myeloma. Multiple myeloma and large B-cell lymphoma were especially linked to obesity in males, whereas cancer of the breast and cervix have been linked to obesity in females. Indeed, approximately 30–50% of deaths caused by breast cancer are due to obesity. There is also an association between obesity and cancers of the digestive tract [20], because obesity-associated hormones and growth factors contribute to the pro-inflammatory environment created by crosstalk between epithelial tumor cells, adipocytes, and inflammatory cells [21]. Specifically, dysregulation of cytokines such as TNF-*α* and interleukin-6 (IL-6) and adipokines such as lectin and adiponectin contribute to the low-grade inflammation that is a hallmark of obesity [22]. Leptin is mainly regulated by insulin-induced changes of adipocyte metabolism and helps to prevent weight gain, whereas adiponectin can increase insulin sensitivity and reduce adipogenic inflammation [23]. Thus, adiponectin can oppose the actions of pro-inflammatory cytokines such as TNF-*α*, IL-6, and monocyte chemoattractant protein-1 (MCP-1) [24].

#### 3.2.1. Clinical Studies on Obesity and Inflammation

 Studies on obese patients show varying results due to differences in study design and the population being analyzed. However, a majority of studies confirm that cytokines and adipokines can regulate obesity-associated inflammation [21–25]. One study found that obese patients have increased concentrations of the C-reactive protein (CRP), the pro-inflammatory cytokine TNF-*α*, and decreased concentrations of adiponectin. However, levels of adiponectin did increase during weight loss, suggesting that weight loss maybe able to restore an anti-inflammatory condition [25]. Strong clinical evidence linking obesity, adipokines, and carcinogenesis comes from a report which found that very obese patients suffering from cancer and/or cardiovascular events had a 30% reduction in mortality following surgery for weight loss [24].

These data on chronic inflammation and abnormal lipid metabolism have important clinical implications because patients are usually diagnosed and stratified on the basis of markers for three separate diseases (type 2 diabetes mellitus, cancer, or metabolic syndrome). In contrast, markers for chronic inflammation and abnormal lipid metabolism are “shared abnormalities” which precede these diseases and therefore allow for earlier diagnoses of these related diseases.

### 3.3. Nuclear Receptors, Lipid Metabolism, Inflammation, and Cancer

 While lipids such as cholesterol and oxidized LDL can play a significant role in tumorigenesis, nuclear receptors are equally important. Nuclear receptors are a unique family of transcription factors which bind DNA and also bind a lipid ligand. Thus, nuclear receptors sense specific lipids and accordingly regulate the expression of specific target genes within adipose tissue. The nuclear receptors regulating lipid metabolism and chronic inflammation are discussed below [26].

#### 3.3.1. Liver X Receptors (LXRs)

 LXRs are cholesterol-sensing nuclear receptors that regulate lipid metabolism and transport and also suppress inflammatory signaling in macrophages by modulating activity of NF-*κ*B [27]. Recent reports suggest that agonistic ligands of LXR suppress proliferation of multiple human cancer cell lines *in vitro* and inhibit the growth and progression of prostate tumor xenografts in nude mice. Notably, phytosterols are agonists for LXRs and are associated with a reduced incidence of colon cancer [28].

#### 3.3.2. Peroxisome Proliferator-Activated Receptors (PPARs)

PPAR*γ* is a transcription factor which regulates insulin sensitivity, adipocyte differentiation, and lipid utilization in adipocytes. In addition to regulating gene transcription, PPAR*γ* binds various lipids (fatty acids, bile acids, and/or sterols) and functions as a major sensor of lipid metabolism. Interestingly, several pre-clinical studies demonstrate that ligands which activate PPAR*γ* receptors (particularly thiazolidinedione derivatives) exert a broad spectrum of anti-tumoral, anti-inflammatory, antiangiogenic, and immuno-modulating activities [29]. The anti-inflammatory effects of PPAR*γ* ligands include stimulation of adiponectin production, which in turn opposes the action of pro-inflammatory cytokines [30]. Indeed, PPAR*γ* nuclear receptors are considered an important component of the molecular pathways interconnecting cancer development with metabolic syndrome [31].

#### 3.3.3. Farnesoid X Receptor (FXR)

Activation of another nuclear receptor, FXR, can also have anti-tumor effects. Thus, FXR deficient mice show increased susceptibility to intestinal tumorigenesis [32] and are more susceptible to inflammation induced by the endotoxin, lipopolysaccharide (LPS) [33]. FXR is a specific bile acid receptor and serves as an important drug target for prevention of colorectal cancer [34], because elevated excretion of secondary bile acids is a strong risk factor for colorectal cancer. Interestingly, guggulsterone's efficacy against hyperlipidemia and its ability to bind FXR also make it a potentially useful drug for colon cancer [13]. Together, these data suggest that normal levels of FXR expression and activity have important anti-inflammatory and anti-tumor effects.

In summary, all three types of nuclear receptors (LXR, PPARs, and FXR) detect signals derived from dietary lipids, pathogenic lipoproteins, or essential fatty acid metabolites and respond by regulating lipid metabolism and suppressing inflammation [26–34]. Since lipid metabolism and inflammation have a major impact on tumor development and progression, drugs which modulate the activities of these nuclear receptors are important anticancer drugs. Having discussed the role of lipid metabolism and inflammation in cancer, we now explain how obesity and diabetes are linked to cancer.

### 3.4. Obesity, Diabetes, and Cancer

 Epidemiological data point to a link between type 2 diabetes mellitus (T2DM) and cancer, which can be independent of obesity. Thus, a large prospective study in the USA conducted a 16-year followup study on a cohort of almost 1million men and women who had no reported history of cancer. The results showed that independent of obesity, T2DM was a strong predictor of mortality from cancer of the colon, pancreas, female breast, male liver, and bladder. In the case of pancreatic cancer, it was unclear whether diabetes was the cause or outcome of pancreatic cancer [20].

The physiological link between obesity, T2DM, and cancer arises, because the adipose tissue in obese individuals produces high levels of free fatty acids, triglycerides, leptin, and pro-inflammatory cytokines. These metabolic changes increase insulin secretion and can lead to insulin resistance which is common in diabetes. Obesity and elevated insulin levels also induce more secretion of insulin like growth factor 1 (IGF-1), which stimulates cell growth and proliferation [20]. A biochemical link between cancer and T2DM exists, because signaling through insulin receptor and insulin like growth factor 1 receptor (IGF-1R) is increased in the hyperinsulinemic condition of diabetics [20]. The hyperactive IGF-1/IGF-IR axis in diabetic individuals can drive proliferation, survival, and growth of tumor cells. Indeed, overexpression of IGF-1R is common in several cancers, and pre-clinical studies show that downregulation of IGF-IR signals can reverse the neoplastic phenotype and sensitize cells to anticancer treatments. Accordingly, several IGF-IR inhibitors have entered clinical trials [35].

 Another biochemical link between cancer and T2DM arises because hyperglycemia generates oxidative stress, which in turn leads to accumulation of modified forms of DNA, protein, and lipids. These modified macromolecules can function abnormally and initiate carcinogenesis. Certain products of oxidative stress, such as advanced glycation end products (AGE), have pro-inflammatory effects. AGE, which consists of glycated, carbonylated, and nitrosylated proteins, accumulates due to aging and diabetes. AGE interacts with its receptor (RAGE) and further enhances oxidative stress and induces inflammation, thus significantly increasing the risk for cancers in diabetic patients [9, 36].

## 4. Diet, Inflammation, and Cancer

### 4.1. Diet, Digestion, and Inflammation

 Thus far, we have explained how the “shared pathology” across obesity, metabolic syndrome, and cancers involves biochemical aberrations in signaling pathways which regulate lipid metabolism and chronic inflammation. However, diet can also contribute to the pathophysiology of metabolic syndrome and cancer. Therefore, this section reviews the evidence linking cancer with diet and inflammation. Many human studies have found high levels of systemic markers of inflammation (high-sensitivity C-reactive protein (Hs-CRP), interleukin-6 (IL-6), and TNF-*α*) in individuals with low-fiber, high-fat diets [37]. A recent study of healthy men and women found that the ratio of omega-6/omega-3 fatty acids showed the strongest positive correlations with increased levels of most inflammation markers, suggesting that this ratio may constitute a predictor of low-grade, chronic inflammation [38].

Although there are several studies on the effects of diet on inflammation markers and the risk of cancer, the influence of digestion on the risk of contracting cancer remains unclear. Studies suggest that the gut microflora (which is influenced by diet and digestion) can influence the pathways linking diet and low-grade inflammation. Thus, fat depots from mice with colitis showed increased expression of inflammatory cytokines and the nuclear receptors PPAR*γ* and FXR [30–33]. Administration of probiotics reversed these pro-inflammatory effects and normalized the gut microflora [39]. Therefore, normal gut microflora potentially have important anti-inflammatory effects. Another interesting study showed that PPAR*γ*, LXR, and FXR are important components of a molecular defense mechanism to protect against accumulation of toxic endogenous lipids and bile acids which accumulate in diet induced hyperlipidemia [40]. As mentioned earlier, drugs modulating FXR activity [32, 33] and guggulsterone [13] have a potential role in treatment of colon cancer, which is associated with fatty diets and elevated secretion of bile.

## 5. Basic Principles of Ayurveda

### 5.1. Doshas, Prakriti, and Disease

Thus far, we reviewed evidence linking inflammation, lipid metabolism, diabetes, and cancer. Before we discuss how ayurveda may provide new biomarkers of chronic inflammation, we explain the basic concepts of ayurvedic physiology.

 Ayurveda defines three dynamic pathophysiological entities (Doshas), as the basis for all body function. The three Doshas are termed as Vata, Pitta, and Kapha, respectively. *Kapha Dosha* governs the nervous and musculo-skeletal systems [41–43]. At the cellular level, Vata Dosha can be associated with signaling pathways regulating cell growth, differentiation, and cell death. Vata Dosha also governs movements of cells, molecules, nutrients, and wastes [44, 45]. The Pitta Dosha is responsible for transformative processes such as digestion, metabolism, energy production, and maintenance of immunity [41–43]. At the cellular level, *Pitta Dosha* can be associated with actions of enzymes, growth factors, hormones, and the reactions required for energy homeostasis and maintenance of basal metabolism [44, 45]. *Kapha Dosha* acts to form and maintain body mass, shape, and flexibility [41–43]. At the cellular level, anabolic processes (such as biosynthesis of macromolecules) and coordination of gene and protein function maybe associated with *Kapha Dosha* [44–46].

In ayurveda, one's basic “body constitution” is termed as “*Prakriti*.” *Prakriti* arises due to a unique combination of fixed amounts of the three *Doshas* at the time of conception. Thus, *Prakriti* determines individuality and is akin to one's genotype. *Ayurveda* recognizes seven main types of *Prakritis*, based on the different combinations of the three *Doshas* at conception. Experimental analysis of the *Prakriti* concept revealed statistically significant correlations between an individual's *Prakriti* and the expression of specific genes and biochemical parameters [44]. Another study found correlations between *Prakriti* and HLA gene polymorphisms [47]. Although one's *Prakriti *(genotype) is fixed, one's *Doshas* are in dynamic equilibrium, and optimal function of each *Dosha* and normal interactions between *Doshas* are essential for good health. Accordingly, individuals with “balanced” *Doshas *(*Sama Prakriti*) are less susceptible to disease than individuals with abnormal *Doshas*. In fact, imbalances or disturbed interactions between *Doshas* are considered a major cause of disease. An abnormal *Dosha* can be inhibited, excessive, or vitiated (disturbed) [48]. Indeed, the type and nature of disease, are primarily determined by the *Dosha* which is affected. For example, inflammatory diseases are associated with vitiation of *Pitta Dosha* [48], whereas obesity and metabolic syndrome are associated with vitiation of *Kapha Dosha *[45, 46]. A specific illness manifests when the vitiated *Dosha(s)* interact with weaknesses in specific organs (*Dhatus*). Conversely, pathogenic factors can also trigger abnormality of the *Doshas* and weaken the *Dhatus* [48]. Severe diseases, such as cancer, affect the entire body and usually involve vitiation of all three *Doshas* [48, 49].

### 5.2. Doshas, Agni, and Immunity

In addition to the concepts of *Doshas* and *Prakriti*, the Ayurvedic concept of *Agni* is important. *Agni* is the primary entity responsible for metabolic and transformative processes at the physiological and cellular levels. There are thirteen types of *Agni* which control all metabolic functions. When *Agni* is strong, digestion of food is normal, and even vitiated *Doshas* can be converted into nontoxic components [50]. “Incompatible foods” (*Viruddha Ahara*) can disturb *Agni* and lead to vitiation* of Doshas*. Indeed, certain useful foods can be pathogenic if ingested in certain combinations or in specific situations. For example, fruits and milk are each useful, but their combination is difficult to digest and can vitiate *Kapha Dosha* and lead to *Agnimandya* (weak *Agni*) [51]. A complex interplay between diet and host factors regulates *Agni* and is in turn influenced by *Agni*. Thus, the nature and composition of diet, quantity of food, timing of food intake, and the intrinsic properties of food are important. In addition, an individual's ability to digest and process food depends on host factors such as *Prakriti*, status of *Doshas*, *Agni*, tolerance, and digestive factors [48, 50, 51]. Thus, a feedback loop mechanism links diet and host factors with the strength and activity of *Agni*. Long-term consumption of incompatible foods can impair this feedback mechanism and increase susceptibility for various metabolic diseases and acute or fatal conditions [48, 50–52]. A weakened *Agni* can also result in decreased immune surveillance, which is a major risk factor for diseases such as cancer. Therefore, maintenance of *Agni* at optimum levels is important for avoiding pathogenesis [50, 51].

### 5.3. Ayurveda and Cancer

Ayurveda does not consider cancer as a distinct disease or set of diseases. Rather, ayurveda states that all diseases result from gross, systemic imbalances and malfunctions of the three *Doshas*. As mentioned above, specific diseases (including cancer) originate from interactions between abnormal *Doshas* and weakened *Dhatus *[48, 49]. For example, vitiation of *Kapha Dosha *is a common link between cancer and diabetes; however, the organs (*Dhatus*) which are affected differ [53, 54]. Thus, weak *Shukra Dhatu *(tissue regeneration and cell division) interacting with vitiated *Vata Dosha* and *Kapha Dosha* could lead to cancer, whereas excess and improperly formed *Meda* (adipose tissue) interacting with vitiated *Kapha Dosha*, can cause diabetes [54]. The magnitude of illness and clinical presentation of cancer are thought to vary, because each person has different patterns of exposure to pathogens and has dynamic changes in the functioning of *Dhatus* [53].

Instead of using targeted therapies for destruction of the tumors, ayurvedic drugs/modes of treatment attempt to correct metabolic defects and restore normal tissue functions (“*Sama Dhatu Parampara*”). Like most forms of traditional medicine, ayurvedic medicine is holistic, since immunotherapy (*Rasayanaprayoga*) for rejuvenating the body's support systems, forms a significant component of cancer therapy [41, 43, 48, 49]. A review of Ayurvedic concepts of cancer and herbal anti-cancer drugs is available in the literature [49].

Table 1 compares the modern and ayurvedic concepts of cancer. It highlights new molecular evidence which validates certain ayurvedic concepts of cancer. Earlier, cancer was thought to result from sequential genetic events regulating cell growth and death. It is now clear that abnormalities involving epigenetic regulation, diet, environmental factors, and immune function significantly affect the phenotype of a cancer patient (Table 1). Ayurveda also considers diet and environmental factors as important regulators of *Agni* and immunity, which in turn can increase risk for cancer. The concept of “shared pathology” between cancer and metabolic syndrome [3, 5, 6] has some similarities to the Ayurvedic view that interactions between vitiated Doshas and weak tissues (*Dhatus*) lead to systemic malfunctions which can manifest as cancers of specific organs (Table 1). As discussed in Sections 2.3 and 3.3, certain anti-inflammatory drugs [11–13] and antidiabetic drugs [29–31] are effective against cancers because of the “indirect” involvement of inflammation and dyslipidemia in carcinogenesis. Ayurveda also uses “indirect” approaches to treat cancers because therapies aim to eliminate vitiated Doshas, rejuvenate body functions, and restore immunity (*Rasayanaprayoga*) (Table 1). Modern, cutting edge, anti-cancer therapy also uses immunotherapy and cancer vaccines.

## 6. The Ayurvedic Concept of “*Ama*” and Intestinal Autointoxication

As mentioned above, abnormal *Doshas*, weakened *Dhatus*, and weakened *Agni* are major risk factors which weaken immune status and predispose an individual to serious diseases such as cancer. We also explained how chronic inflammation, a hallmark of carcinogenesis [5–7], is associated with poor diets [37–39], abnormal lipid metabolism [9, 18–33], *Kapha Dosha* [45, 46], and metabolic syndrome [20–24]. We now discuss the Ayurvedic concept of “*Ama*” since it pertains to the origin of chronic inflammation.

“*Ama*” is a toxic, heavy, unctuous, and sticky juice which originates as a waste-product of digestion and metabolism. Indeed, the word “*Ama*” can be translated to mean “immature” or “incompletely digested.” “*Ama*” builds up in individuals whose digestion is either weak or overloaded with the wrong foods [55]. Since one's digestive powers (*Agni*) are in part determined by one's *Prakriti* (genotype), individuals with strong digestive powers (a characteristic associated with *Pitta Prakriti*) can eat larger quantities and richer foods without forming “*Ama*.” In contrast, individuals with weak *Agni* have weak digestive powers (a characteristic associated with *Kapha Prakriti*) and produce “*Ama*” more easily. Ayurveda states that simple foods minimize formation of “*Ama*,” whereas foods with high protein or fat content result in increased production of “*Ama*” because such foods are intrinsically difficult to digest and are therefore more likely to be partially digested [55]. Food or water consumption before complete digestion of previously consumed food also causes “*Ama*” and leads to vitiation of all three *Doshas*. Overall, a weakened *Agni* is the root cause of “*Ama*,” which is a major risk factor for disease [56].

The Ayurvedic concept of “*Ama*” is similar to the Egyptian concept of “*Ukedu*,” and the old theory of intestinal auto-intoxication propounded by *Metchnikoff* [48]. Thus, *Metchnikoff *believed that proteolytic gut bacteria can produce toxic byproducts (phenols, indoles, and ammonia), from digestion of dietary proteins. These toxic byproducts of digestion accumulated with age and caused disease [57]. Interestingly, modern evidence supports *Metchnikoff*, since bacterial species which metabolize dietary carcinogens (heterocyclic amines) from cooked meat and fish are associated with increased risk of tumors [58]. The link between intestinal auto-intoxication and disease is concordant with Ayurvedic concepts on “*Ama*” and its pathogenic potential (please see the next two sections).

### 6.1. Diagnoses and Treatment of “*Ama*”

 An ayurvedic practitioner diagnoses “signs and symptoms” of “*Ama*.” Typically, “*Ama*” manifests as a sticky, white coating on the tongue, which obstructs various internal microchannels and is associated with characteristic symptoms such as inability to taste food, local or general inflammation, sudden fatigue, heaviness, pain, abdominal discomfort, lethargy, indigestion, and constipation [48, 59]. Since “*Ama*” is considered the root cause of disease, it must be fully digested before one can rectify vitiated *Dosha(s)* [59]. Accordingly, strengthening of *Agni* and complete digestion of “*Ama*” are major goals of ayurvedic treatment. Thus, therapies such as purgation, enema, or therapeutic emesis lead to complete digestion of “*Ama*” and separation of the vitiated *Dosha* from various channels [55]. Improved *Agni* results in complete digestion of “*Ama*,” and causes the condition known as “*Nirama*” (free from “*Ama*”). The “*Nirama*” stage is favorable for additional treatments aimed at resolving vitiated or depleted *Dosha* and *Dhatus* and reversing the disease pathology[55]. Notably, the popular *Panchakarma* procedures for eliminating vitiated *Doshas* are only effective if preexisting “*Ama*” has been completely digested [55].

### 6.2. “*Ama*” and Pathogenesis

The obstruction of micro-channels by “*Ama*” is responsible for loss of homeostasis, inflammation, and tissue damage [60]. Accordingly, ayurveda believes that “*Ama*” is the root cause of several diseases since it blocks important micro-channels (*Srotas*) which nourish tissues (*Dhatus*) [61]. Being a sticky substance, “*Ama*” is usually conjugated with *Doshas *or *Dhatus*. For example, *Pitta Dosha* in conjunction with “*Ama*” is termed as “*Sama Pitta*,” which can trigger pathogenesis and hamper physiological functions [50]. Excessive “*Ama*” can circulate and interact with excretory products to produce a reactive and toxic form with antigenic and pro-inflammatory properties. This form of “*Ama*”can potentially disrupt the immune system and increase severity of the initial disease [48]. In this context, it is intriguing to note that modern science also finds that chronic inflammation may be caused by nondigestible particles [11].

### 6.3. Experimental Approaches for Investigating “*Ama*”

In the context of cancer and inflammation, it is important to study “*Ama*” because it is believed to have antigenic and pro-inflammatory properties [48]. Pre-clinical experiments can be done to determine if “*Ama*” can serve as a reliable, early biomarker of chronic inflammation. Isolation of pure “Ama” maybe problematic because it is primarily localized in micro-channels of the body. According to Ayurveda, excess “*Ama*” can be found on the tongue and in urine. Therefore, it is important to properly identify and collect samples of tongue secretions and urine after “clinical” characterization and confirmation of “*Ama*” stages of disease as described in Ayurveda [48]. If available, one should use samples from the same individual prior to disease (“*Nirama*” stage), as controls. These paired samples can then be tested for potential toxicity, immunogenicity, and pro-inflammatory activity, *in vitro*, and in appropriate animal models. This can be done by adding different concentrations of “*Ama*” containing samples versus “*Nirama*” controls, to cell lines derived from various tissue types. One can then determine if “*Ama*” is cytotoxic by doing MTT and/other assays for cell viability and cell death (apoptosis and necrosis assays) [62]. Since most cell lines are immortalized or transformed, it is also important to assess the potential toxicity of “*Ama*” on primary cultures derived from normal tissues. If “*Ama*” is cytotoxic *in vitro*, these toxicity experiments should also be replicated in animal models.

In order to determine if “*Ama*” can induce inflammation, one can add different concentrations of “*Ama*” containing samples versus “*Nirama*” controls, to different cell types and measure various pro-inflammatory molecules (cytokines, adipokines, eicosanoids, NF-*κ*B, and STAT-3) [5–7, 21–25]. If these results suggest that “*Ama*” has pro-inflammatory properties, one can inject different concentrations of “*Ama*” containing samples versus “*Nirama*” controls, into separate groups of mice, and measure these various pro-inflammatory molecules [5–7, 21–25] and inflammation markers (CRP and ratio of omega-6/omega-3 fatty acids) [22, 24, 37, 38]; in experimental animals versus controls. If these results also show that “*Ama*” containing samples induce inflammation when compared with “*Nirama*” controls, then the concept of “*Ama*” as a pro-inflammatory molecule would be proved.

### 6.4. Candidate Molecules for “*Ama*”

 If the above experiments show that “*Ama*” containing samples can trigger chronic inflammation, one can comparatively analyze the biochemical composition of “*Ama*” versus “*Nirama*” samples. One should be also able to compare the biochemical composition of “*Ama*” with various endogenous, pro-inflammatory molecules produced during digestion, metabolism, and energy production. There are at least five types of endogenous pro-inflammatory molecules which may represent “*Ama*”. First, improperly digested foods in obese individuals are excellent candidate molecules for “*Ama*”, because they are associated with altered composition of gut microflora [37, 39, 57, 58], which in turn can induce chronic inflammation through activation of the lipopolysaccharide toll-like receptor-4 axis [11, 63]. The link between high fat diets, increased secretion of bile acids (such as deoxycholic acid), and increased risk for colon cancer is known [28, 34]. However, a direct link between bile reflux, inflammation, and cancer comes from a study showing that unconjugated bile acids potently stimulate expression of cyclooxygenase-2 (COX-2), a major pro-inflammatory enzyme in oesophageal adenocarcinoma-derived cells [64]. Thus, specific bile acids can have a pro-inflammatory nature and therefore represent a second molecular candidate for “*Ama*” [34, 64]. The pro-inflammatory nature of abnormal bile acids is also suggested by the fact that modulation of bile acid receptor (FXR) has anti-inflammatory effects (Section 3.3). The third molecular candidate for “*Ama*” represents advanced glycation end-products (AGE) which accumulate in diabetes, ageing, and cancer. AGE may represent “*Ama*,” because interaction of AGE with its receptor (RAGE) triggers chronic inflammation [8, 9, 36]. The fourth molecular candidate for “*Ama*” consists of protein-lipid peroxide (P-LPO) adducts, since LPO (which accumulate during oxidative stress) can form adducts with lysine residues of certain proteins and induce the expression of the pro-inflammatory cytokine, TNF-*α* [65]. Lipid peroxides can also react with DNA to produce promutagenic adducts such as etheno-deoxyadenosine. Increased levels of such etheno-DNA adducts in human blood and urine, may serve as biomarkers of chronic inflammation in individuals who are prone to cancer due to prior exposure to carcinogens [66]. Hence, such etheno-DNA adducts could represent a fifth molecular candidate for “*Ama*.”

The above experiments may reveal that “*Ama*” is enriched in one of these five candidate molecules. On the other hand, “*Ama*” may consist of a heterogenous mixture of these five candidate pro-inflammatory molecules (partially digested food particles, specific bile acids, AGE, peptide-peroxidized lipid adducts, and etheno-DNA adducts) derived from abnormal digestion and metabolism in organs and cells.

### 6.5. Potential Clinical Significance of *Ama*

 According to ayurveda, excess “*Ama*” can be found on the tongue, and in urine [48]. Therefore, comparative and quantitative biochemical analysis of “*Ama*” from healthy individuals versus patients suffering from different cancers, metabolic syndrome, or both diseases can be done. In addition, one could measure the levels of pro-inflammatory markers and degree of immunosuppression in patients with different cancers and in patients with metabolic syndrome and a specific type of cancer. Such clinical studies may find statistically significant correlations between the biochemical composition and levels of “*Ama*,” and the severity of metabolic syndrome and/or cancers. Since Ama' originates from improper digestion and metabolism in normal, non-obese, individuals [48, 55, 56] it should be detectable at early stages. Accordingly, increased levels of “*Ama*” may well precede the expression of the major pro-inflammatory molecules discussed above. If levels of “*Ama*” (as diagnosed by Ayurveda) significantly correlate with the levels of one or more of the five candidate molecules mentioned above, then “*Ama*” may prove to be a reliable biomarker of early inflammation in patients at risk for metabolic syndrome and/or cancers. If “*Ama*” can serve as a reliable biomarker of early inflammation, it may lead to identification of a new sub-type of patients with digestive disorders, inflammation, and high risk for cancer and metabolic syndrome. Such a discovery could have enormous clinical significance, because it implies that “wellness therapies” (diet and life-style changes, detoxification therapies) may lead to reduced “*Ama*,” and prevent onset of chronic inflammation and its associated diseases (cancer and metabolic syndrome).

In summary, the pre-clinical experiments and clinical experiments outlined above can determine if “*Ama*” is a reliable biomarker of early inflammation. Although C-reactive protein, TNF-*α*, and IL-6 are well-established inflammation markers associated with high fat diets, these markers reflect later stages when immune dysfunction has already occurred [22, 24, 37]. Similarly, the ratio of omega-6/omega-3 fatty acids [38] and AGE [8, 9, 36] are highly reliable inflammation markers for dietary status and levels of oxidative stress, respectively. It is estimated that 80% of cancer in USA have a nutrition/diet component suggesting a great impact of functional food and food components on incidence and treatment of cancer [67]. Currently, no single marker is available to predict the outcome of a dietary intervention on the resistance to infection or to other immune-system-related diseases [68]. If “*Ama*” proves to be a reliable biomarker of early inflammation, it could potentially link inflammation with improper diet and digestion. Thus far, altered gut microflora represent the only biomarker linking digestive status with inflammation [39, 57, 58]. There are no established biochemical markers linking weak/abnormal digestive and metabolic status with inflammation. “*Ama*” may well prove to be this missing “biochemical marker.”

## 7. Conclusions

In the introduction we cited a review which warned that advances in personalized medicine may only occur with incremental improvements, due to lack of biomarkers for patient stratification. One way to avoid this scenario is to develop innovative concepts and approaches in the fields of translational and personalized medicine, by tapping into the wisdom of traditional systems of medicine. Thus far, research on ayurveda has focused on identifying and validating the bioactivities of phytochemicals isolated from various medicinal plants. However, ayurveda and other traditional systems of medicine are not merely sources of “raw materials” for potential drug candidates. Rather, the principles and practices of traditional systems of medicine could be developed into hypothesis which can be tested by modern scientific methods. This approach may provide evidence which validates some traditional concepts and may lead to the development of novel biomarkers for wellness and disease.

Evidence from the omics revolution and systems biology clearly point to a strong degree of connectivity between physiological and molecular pathways that were considered independent. For example, the newly discovered links between inflammation, lipid metabolism, and cancer were unexpected. Therefore, this paper explains and analyzes the links between two major diseases (cancer and metabolic syndrome). These new findings vindicate the holistic approach of ayurveda and other traditional systems of medicine, because it proves that a disease cannot be considered as a sequence of defective genetic and biochemical steps. Indeed, the links between inflammation, metabolic syndrome, and cancer suggest that even seemingly distinct diseases can arise from fundamental aberrations in metabolism, homeostasis, and immune function. Thus, the advances in omics analysis and systems biology are providing concrete evidence for some of the holistic concepts in traditional systems of medicine. If diseases are diagnosed and analyzed in a holistic manner, then treatment of disease is also holistic. Accordingly, ayurvedic drugs/treatment regimens are largely designed to restore the body's natural defense mechanisms and self-healing powers. These therapies are aimed at ensuring long-term recovery from disease by strengthening and rejuvenating major body systems. This holistic approach of ayurveda is also true of other traditional systems of medicine and is precisely what attracts people to alternative medicine. Indeed, we are in an exciting phase of modern medicine, wherein rigorous scientific evidence supports some aspects of holistic, traditional medical systems. Sustained and collaborative efforts between ayurvedic physicians, clinicians, and basic sciences researchers may lead to a deeper understanding and even convergence of certain modern and traditional principles underlying health and disease.

## Figures and Tables

**Table 1:** 

Medicines' earlier concepts of cancer	Ayurvedic concepts of cancer	Evidence supporting ayurvedic concepts of cancer
Cancer results from sequential genetic events which lead to uncontrolled cell growth and resistance to cell death.	Cancer results when abnormal interactions between *Prakriti *(genotype) and environmental factors vitiate the *Doshas* and impair immunity.	Abnormalities besides aberrant cell growth and cell death cause cancer. Epigenetic regulation, diet, environmental factors, and immunity affect phenotypes.

Most cancers arise due to sporadic mutations in specific tissues, and spread to other organs.	Interaction between vitiated *Doshas* and weak tissues (*Dhatus*) manifests as cancers of specific organs.	Shared molecular pathology between cancer and metabolic syndrome.

High-fiber diets associated with lower risk of heart disease and cancer. Inflammation process was not linked to cancer.	Links between improper diet, digestion, metabolism, inflammation, and disease. “*Ama*” maybe a novel biomarker for early inflammation.	Chronic inflammation actively promotes all stages of carcinogenesis.

Chemotherapy or radiotherapy are not selective for cancer tissue. These therapies also destroy normal tissue and have severe side effects.	Therapies indirectly target cancer tissue by eliminating vitiated *Doshas*, rejuvenating *Dhatus*, and restoring immunity.	Anti-inflammatory and antidiabetic drugs indirectly destroy cancer tissue. Immunotherapy. Cancer vaccines.
